# Perceived value and its relationship to satisfaction and loyalty in cultural coastal destinations: A study in Huanchaco, Peru

**DOI:** 10.1371/journal.pone.0286923

**Published:** 2023-08-01

**Authors:** Otto Regalado-Pezúa, Mauricio Carvache-Franco, Orly Carvache-Franco, Wilmer Carvache-Franco

**Affiliations:** 1 ESAN Graduate School of Business, Universidad ESAN, Lima, Peru; 2 Universidad Espíritu Santo, Samborondón, Ecuador; 3 Facultad de Economía y Empresa, Universidad Católica de Santiago de Guayaquil, Guayaquil, Ecuador; 4 Facultad de Ciencias Sociales y Humanísticas, Escuela Superior Politécnica del Litoral, ESPOL, Guayaquil, Ecuador; The University of Tokyo, JAPAN

## Abstract

Coastal tourism offers a wide variety of activities related to nature and culture in a sustainable environment. The present study in a coastal destination with cultural characteristics aims to (i) establish the dimensions of perceived value, (ii) determine the relationship between perceived value and satisfaction, and (iii) identify the relationship between perceived value and loyalty in variables such as return, recommendation, and word of mouth in a cultural coastal destination. This quantitative research used a sample of 384 valid questionnaires collected in Huanchaco, Peru, a city next to the Pacific Ocean, being a coastal destination with cultural potential. Factor analysis and multiple regression were applied for data analysis. The results show three dimensions of value perceived by tourists in coastal destinations: emotional and social value, economic value, and functional value. Of these, emotional and social value is the most salient predictor of tourist satisfaction and loyalty. These results will serve as management guides for cultural coastal destination managers and contribute to the academic literature.

## 1. Introduction

After the COVID-19 pandemic, people will look for adventure trips, natural spaces, and safe and quality experiences [1]. Therefore, it poses a great opportunity for coastal and marine tourism to create tourist spaces with biosecurity measures suitable for visitors, despite the enormous challenges of balancing environmental problems with tourism activities [2]. Coastal tourism refers to activities based on coastal lands, such as swimming, surfing, sunbathing, and other coastal leisure, recreation, and sports activities on the shores of a sea, a lake, or a river. Proximity to the coast is a condition for services and facilities supporting coastal tourism [3]. Likewise, coastal tourism can be understood as part of marine activities since both are closely linked. Maritime tourism includes activities at sea, such as cruises, sailing on yachts or boats, and water sports and their respective services and infrastructures on land [3]. Hence, coastal and marine destinations can offer a wide range of tourist activities such as visiting local communities, practicing water sports, tasting the local cuisine, sightings of marine flora and fauna, and ecotourism [4].

Regarding coastal tourism demand, the study of perceived value is crucial for the sustainable development of destinations. Perceived value is subordinated to the judgments of the tourist, whose evaluation of the results involves the information prior to the purchase, the quality of the services, the tourist resources, the surrounding nature, time, money, and effort invested, among other aspects [5]. In this way, perceived value has been considered a reliable concept to anticipate tourist behaviors [6, 7]; it is closely related to consumer behavior as it is a predictor of behavioral intentions [8, 9]. Therefore, perceived value leads to favorable outcomes such as satisfaction and behavioral intentions [10, 11]. For this reason, the concept of perceived value has been widely used to analyze and understand the future behavior of tourists concerning purchase decisions [5].

In this context, the city of Huanchaco is a coastal destination located in Peru, which offers a wide variety of activities, such as windsurfing, kitesurfing, and surfing, both as a pastime and competitively with a world surfing circuit, The Longboard Pro World Championship, held every summer. In the same way, the beach and the boardwalk are used for outdoor sports such as jogging, walks, bicycle rides, and yoga in the sand, in groups and individually.

Within this order of ideas, the literature on perceived value in coastal destinations is still scarce, so it is crucial to carry out this type of study as a contribution to sustainable development. Therefore, our research in the cultural coastal destination of Huanchaco, Peru, has the following objectives: (i) establish the dimensions of perceived value, (ii) determine the relationship between perceived value and satisfaction, and (iii) identify the relationship between perceived value and loyalty in variables such as return, recommendation and word of mouth in a cultural coastal destination. Being the city of Huanchaco a coastal destination with potential for cultural tourism, it will be a novel study different from other previous findings. The results will serve as management guides for managers of coastal destinations and contribute to the academic literature.

## 2. Literature review

### 2.1. The perceived value in coastal destinations

For some scholars, perceived value is primarily based on a utilitarian perspective, whereby economic and cognitive valuations are employed to examine the mental trade-off between costs and benefits/quality [12]. However, other authors such as Chi & Kilduff [13], Koller et al. [14], and Lee et al. [15] argue that a utilitarian perspective is too narrow and simplistic to encompass holistic representations of value perception as an intrinsic dimension. Therefore, it is recommended that perceived value be based on a multidimensional construct containing an emotional value, social value, and hedonic and utilitarian dimensions that critically construct positive emotions and customer satisfaction [15].

Perceived value is recognized as a multidimensional concept, which involves individual evaluations of the benefits obtained in the travel experience, compared to the sacrifices made and conditioned by rational, affective, and social nature aspects [16]. Furthermore, the concept of perceived value has been widely used to analyze and understand the future behavior of tourists concerning purchasing decisions [5]. Consequently, the emphasis on perceived value also provides a good basis for attracting responsible tourists who share common values [17].

Previous findings on perceived value in coastal destinations have focused on different perspectives. Williams and Soutar [18] identified five dimensions of perceived value in an adventure context on the Australian coast: functional, emotional, social, novelty, and value for money. They determined that all these dimensions significantly influence tourist satisfaction. Jamal et al. [19] established five dimensions of perceived value in Malaysia: functional value (establishment), functional value (price), experimental value (host-guest interaction), experiential value (activity, culture, and knowledge), and emotional value. From a more sporting perspective, Schoeman et al. [20] researched the perceived value of a diving experience in Sodwana Bay, South Africa. They found five perceived values: perceived emotional value, perceived risk value, perceived functional value, perceived social value, and perceived epistemic value. The epistemic value was rated as the most important for divers in the marine environment.

From an ecotourism perspective, Kim and Thapa [21] found four perceived values on Jeju Island in South Korea, namely quality, emotional, price, and social. The authors indicated that perceived quality, emotional and social values significantly affect flow experience and satisfaction. In addition, flow experience was significantly and positively related to satisfaction, environmentally responsible behaviors, and destination loyalty. In a study conducted in the coastal city of Lima, Peru, Carvache-Franco et al. [22] found two dimensions of perceived value: economic-functional and emotional-social. These two dimensions were also predictors of tourists’ satisfaction and loyalty. Finally, Carvache-Franco et al. [23] found three dimensions of perceived value in Costa Rica, namely emotional and functional value, social value, and economic value.

We have been able to analyze that destinations, because they have different attractions, can vary their perceived value as we have seen in previous findings. For example, in ecotourism four perceived values have been found, namely economic and functional value and social value [17]. In the same way, in another ecotourism study, Quality, emotional, price and social were found as dimensions of perceived value [21]. Therefore, the perceived value construct may vary according to the type of tourism and according to the attractions of the destination.

In summary, several previous studies have addressed perceived value in coastal destinations, considering different contexts, such as adventure, sports, and the environment. In addition, some recurrent dimensions of perceived value have been found in these destinations, such as functional, emotional, price, economic and social. However, there is still little literature on this subject in coastal destinations with cultural characteristics, so our first research question arises.

RQ1: What are the dimensions of perceived value in cultural coastal destinations?

### 2.2. Perceived value, satisfaction, and loyalty in coastal destinations

Future consumer behavior is a product of the perceived value of each tourism experience [16]. Several studies have established that perceived value exerts a positive and significant effect on experience satisfaction [24, 25]. As for previous findings on perceived value and the relationship with satisfaction and loyalty in coastal destinations, Jin et al. [26], in a water park in South Korea, found that the perceived value and image of the park exert a direct influence on customer satisfaction, and also positively affect behavioral intentions. In a study on Green Island (Lyudao in Chinese), Taiwan, Cheng and Lu [27] determined that destination image leads to a greater perception of novelty, promotes hedonic and perceived value, and encourages tourists’ revisiting behavioral intention. In Bangladesh, Hasan et al. [28] revealed that both the quality of service and perceived values directly affect destination image, attitudes, and tourist satisfaction. Frías Jamilena et al. [29] analyzed a sample of 503 British tourists visiting Spain and found a high self-congruence between the tourist and the destination, contributing significantly to the perceived value of the destination. They also identified that tourist motivations contribute considerably to destination-value creation and that the previous experience of the tourist is a critical moderator of destination value-formation.

Lee et al. [30] analyzed the effect of the perceived value of ecosystem services on tourists’ intentions to revisit the Aogu Coastal Wetland in Taiwan. They found that the perceived value of wetland ecosystem services positively affects tourists’ environmental concerns and friendly environmental behavior. In Bangladesh, Hasan et al. [31] established that tourist attitude to visiting behavior mediates the relationship between destination image, perceived value, satisfaction, and behavioral intention significantly, while it has no mediating effect between perceived risks and behavioral intention.

Du et al. [32], in the Shenzhen Mangrove Nature Reserve, a coastal city in China, found that perceived value contributes the most to environmentally friendly behavior, and tourism satisfaction mediates the relationship between environmental knowledge and environmentally friendly behavior. In the context of marine tourism in Zhoushan, China, Su et al. [33] revealed that object-based authenticity and interpersonal authenticity significantly affect the perceived value and loyalty of tourists, and perceived value partly mediates the relationship between authenticity and loyalty. For Carvache-Franco [22], the economic-functional dimension of perceived value is the most important predictor of satisfaction, and the emotional-social dimension is the most significant predictor of loyalty in coastal and marine destinations.

In Costa Rica, Carvache-Franco et al. [23] also found that the emotional and functional dimension is the most important predictor of satisfaction and loyalty variables, such as return, recommendation, and word-of-mouth intentions about a coastal and marine destination. For Tkaczynski et al. [34], whale watchers’ interest in protecting the environment was a significant predictor of perceived quality and emotional value. Perceived value mediated whale watchers’ satisfaction and interest in preserving the environment.

In summary, several previous findings have addressed a relationship between perceived value, satisfaction, and loyalty in coastal tourism. In addition, other studies have analyzed the dimensions of perceived value that influence satisfaction and loyalty in coastal tourism. In this perspective, for some academics, all the dimensions influence satisfaction and loyalty, while for others, there are more predominant ones, such as functional, social, and emotional. However, to date, no studies have shown the dimensions of perceived value that predict satisfaction and loyalty in coastal destinations. If we find out which are the dimensions of the perceived value that predict satisfaction and loyalty, we can create guidelines to increase these factors to improve the tourist’s stay in coastal destinations with cultural potential. Therefore, our second and third research questions arise.

RQ2: What dimensions of perceived value predict demand satisfaction in cultural oastal destinations?
RQ3: What dimensions of perceived value predict loyalty variables such as return, recommendation, and word-of-mouth intentions about cultural coastal destinations?

## 3. Methodology

### 3.1. Study area

The city of Huanchaco is located on the north coast of Peru, specifically in the province of Trujillo, in the department of La Libertad. It is located 13 km from the center of the city of Trujillo, so in addition to the tourists, it facilitates the visit of the inhabitants of this nearby city on excursions on weekends. This proximity is a great advantage for this coastal destination since the flow of visitors positively influences its urban and tourist development [35].

Huanchaco was selected as one of the main surfing beaches in Peru. The quality of the waves in the north of the country and the practice of various international championships confirm this. In addition, its proximity to the city of Trujillo, known as the capital of eternal spring of its excellent weather all year round, means that the beach is frequented all year round by surfers but also by visitors who enjoy contact with nature and the local gastronomy, especially based on fish. In the same way, the proximity to pre-Inca archaeological remains such as Chan Chan makes the place a mandatory visit mainly for domestic tourists. This permanent flow of tourists and visitors allows Huanchaco to offer tourist services throughout the year and not only during the summer.

After presenting the area of study and the justification for its selection, we will go deeper into it considering different contexts, such as geographic, cultural, environmental, and business contexts, as well as the practice of coastal and adventure sports, which will allow us to understand better the dimensions of the value perceived in Huanchaco and its surroundings. Likewise, this description will enable an understanding of how the offer and service levels, in general, the integral value proposition of Huanchaco, contribute to the satisfaction of tourists and visitors, as well as their loyalty to return and recommend the destination. For all these reasons, the city of Huanchaco is a coastal destination with ideal cultural attractions for our study that may contribute contributions to the academic literature in original findings other previous studies have not published. To answer the research questions, it was necessary to apply the present model of perceived value in a study area that has potential in coastal tourism and culture, therefore, the city of Huanchaco is the right destination to answer these questions of our studio.

Huanchaco is divided into nine districts: El Milagro, Valdivia, Huanchaquito Alto, Santa María, Villa del Mar, Huanchaquito Bajo, Pampas de Alejandro, and Cerrito de la Virgen. Its total population is 68409 inhabitants [36]. According to Quispe Del Águila [35], Huanchaco has been inhabited by Moche and Chimú culture communities since pre-Columbian times. This ancestral community is fortunate to consist of families with Chimú traditions that still preserve their customs and traditions: artisanal fishing in a *totora* horse, their gastronomy, and their beliefs and festivities.

One of the most representative pieces of this coastal culture is the *caballito de totora*. It is a boat (kind of raft) made of *totora*, a type of light reed that develops abundantly in humid places. The Chimú produced these horses and used them as a means of transport for one or more people; it was also used for fishing activities and the extraction of guano from the islands. This vessel could carry up to 100 kg [37].

According to Cayetano Minchola [38], Huanchaco has significantly developed since the nineties, when it began to offer tourists and visitors complementary services, such as travel agencies, hotels, traditional restaurants, transport companies, recreation, sale of handicrafts, and leisure activities. However, this development has also presented weaknesses in tourism infrastructure and threats to its sustainability. For example, the destination lacks good environmental management to control the predation of the *totorales*, the raw material for the manufacture of the *totora* horses, which are used by artisanal fishermen and also as a tourist activity.

**Fig 1:**
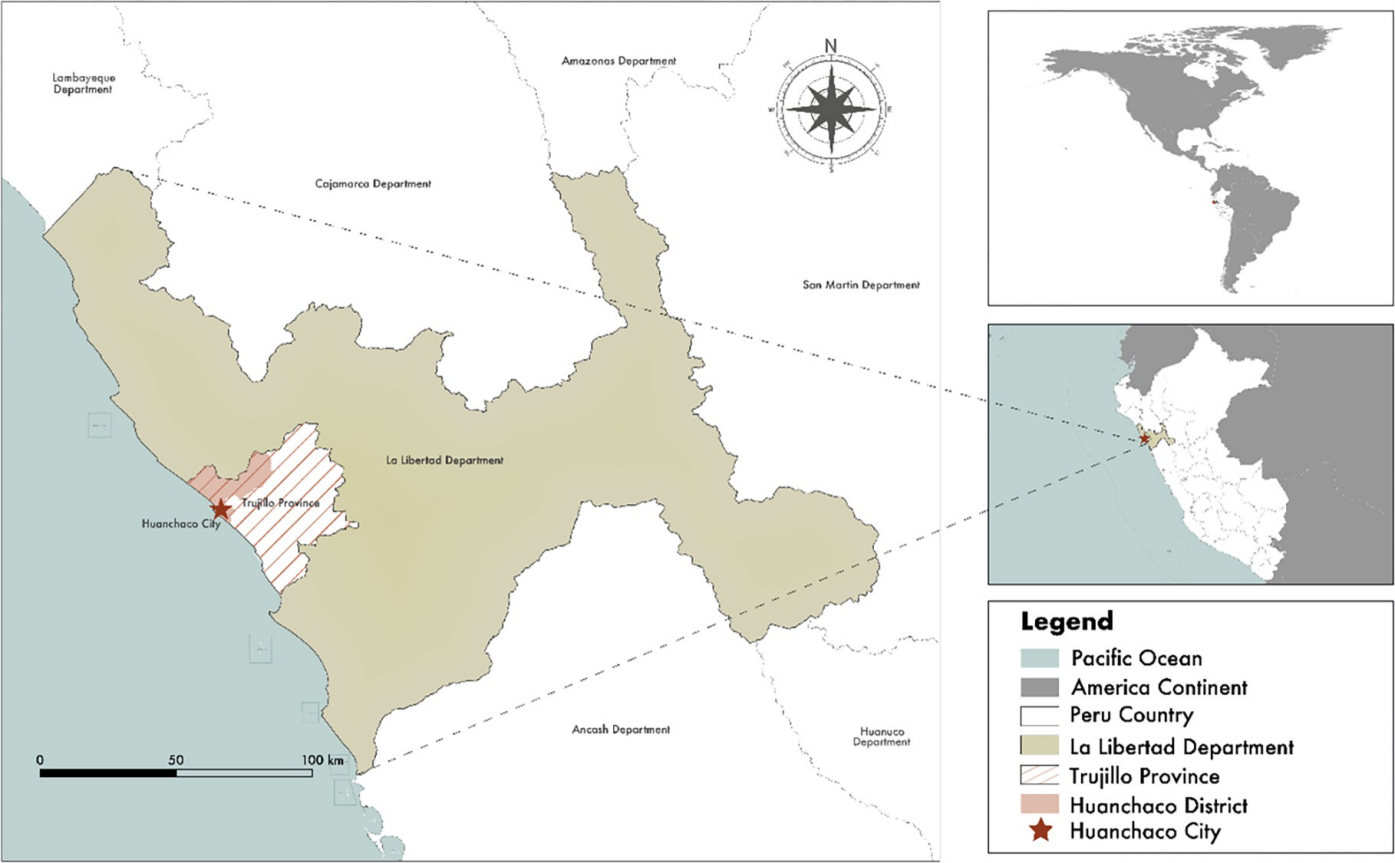
Geographic location of the city of Huanchaco, Peru. **Resources:** Authors. The figure is similar but not identical to the original image and is therefore for illustrative purposes only. Used programs: Q-GIS, version 3.28. ArcGIS, version 10.8. Shapefile sources: Basemap: OpenStreetMap. Zoning maps of the country: National Institute of Statistics and Informatics of Peru (INEI, 2022), it is a publicly accessible information base.

In this regard, the beaches of Huanchaco offer rides in *totora* horses. The Wachaque Chimora organization monitors the safety of people who wish to enjoy these rides. This organization promotes the revaluation of the *totora* horse, the strengthening of cultural identity, and above all, the improvement of the quality of life of artisanal fishermen. In addition, it makes sure that the associates comply with the established security measures and keep the beach clean. All this aims to improve travelers’ experience in the city [38]. (Fig 1).

Another tourist attraction of the city is its church. It is the second oldest church in Peru and was built in 1540 in a Huaca of the Chimú culture. Inside is the Virgen del Socorro image, considered Huanchaco’s patron saint. Also, it has an artisan pier built in July 1891 and used in the commercial boom that the city had until 1914. Currently, it is a tourist pier that was remodeled as an attraction to encourage tourism in the city. The entrance arch to the pier is made of wood with fine finishes and has in its design an iconography of the Chimú culture [39].

After the paralysis of tourist activity due to the COVID-19 pandemic, the city obtained the sanitary certification of Safe Travels with the purpose of receiving around 45,000 tourists every weekend during the summer of 2022; the average was around 12000 [40].

This proves that the authorities of Huanchaco are constantly investing in the city, remodeling it, and creating new spaces [41]. They invested about USD 150,000 in the Parador Turístico "Quibisich," which has a wooden sculpture of the Chimú culture and carefully considered details, such as clay reliefs characteristic of this culture, another set of reliefs similar to those found in Chan Chan, and the Diorama of elite Chimú characters.

Surfing is another coastal activity that can be done in Huanchaco, both as a sports practice and at a competitive level, due to the characteristics of the waves in the north of the country. According to Huet [42], Huanchaco is an important surfing destination in Peru, where several international competitions are held every summer, including the Longboard Pro World Championship. Therefore, there is a greater offer of schools with the rental of wetsuits and the practice and the teaching of surfing to tourists.

**Fig 2:**
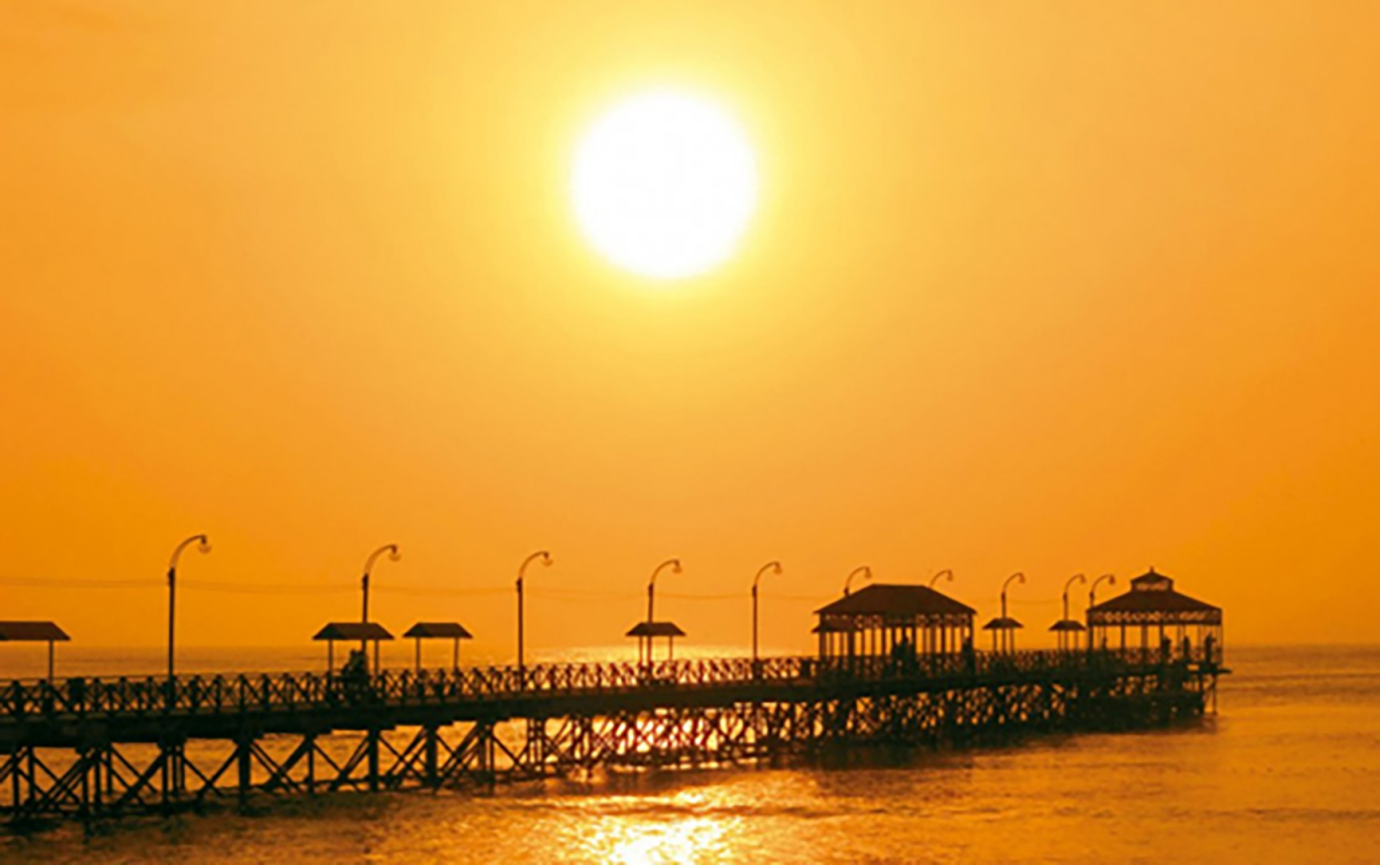
Huanchaco craft dock. **Resource:** https://munihuanchaco.gob.pe/atractivo-detalle.php?id=27.

**Fig 3:**
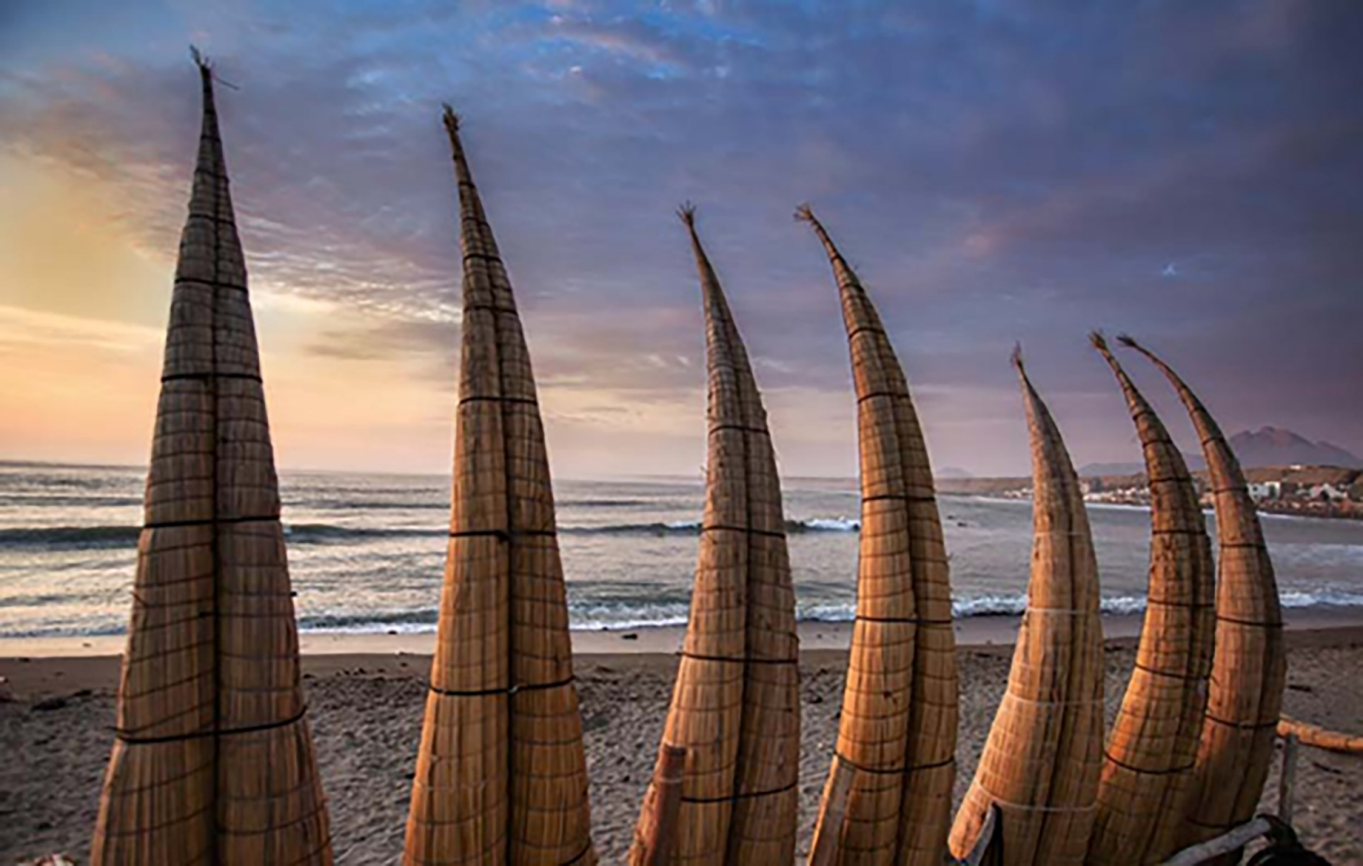
The Caballito de Totora. **Resource:** https://www.peru.travel/pe/masperu/caballitos-de-totora-milenaria-embarcacion-para-la-pesca-en-peru-que-aun-se-mantiene.

In addition to surfing, other water sports practiced are windsurfing and kitesurfing. Finally, the beach and the boardwalk are used for outdoor sports such as jogging, hiking, cycling, and yoga in the sand [43]. (Figs 2 and 3).

### 3.2. The survey, data collection, and analyses

The present study set the following objectives: (i) establish the dimensions of perceived value, (ii) determine the relationship between perceived value and satisfaction, and (iii) identify the relationship between perceived value and loyalty in variables such as return, recommendation, and word of mouth. To this end, a questionnaire consisting of three parts was designed. The first section contained eight closed-type sociodemographic questions adapted from the study by Lee et al. [44]. The second part evaluated the perceived value of a coastal destination and was made up of 12 items adapted from the study of Kim and Park [17] and Carvache-Franco et al. [22]. These questions were measured on a 5-point Likert scale (1 being not important and 5 very important). Through this part of the questionnaire, the dimensions of the perceived value of this study were found. These dimensions were selected through an exploratory factor analysis. This factorial analysis found the dimensions of the perceived value according to the correlations of the items in each factor.

The third section measured overall satisfaction with Likert-type questions, where 1 was not very satisfied, and 5 was very satisfied. Finally, loyalty was measured with three Likert scale questions, ranging from 1, unlikely, to 5, very likely, adapted from Kim and Park’s study [17].

The Cronbach’s Alpha index was used to determine the reliability of the perceived value scale, which reached the value of 0.93, indicating a high consistency between the elements of the scale. Among the aspects that were adapted was proposing a scale that encompasses the attractions of a coastal destination with cultural characteristics and that would be understandable in this region of South America. The questionnaire was analyzed by three experts in the field who confirmed its validity as a measuring instrument. The novelty of this paper is that it proposes a perceived value construct applicable to cultural coastal destinations that has not been analyzed in previous findings.

The sample was collected in situ in the coastal city of Huanchaco, Peru, during August and September 2022. The interviewers were trained by the authors of the present study. The convenience sampling method was used since tourists were selected for convenient accessibility and proximity to the interviewers. Respondents had to be 18 years or older at the time of answering the questionnaire, and the survey initially contained informed consent. The study was approved by ESPOL Polytechnic University in Ecuador. A pilot test was conducted first with ten people on the first day of the study. Once the corrections were made, the surveys were collected. The corrections had to do with the wording of the questions and their location in the questionnaire.

Using the sample with an infinite population, a margin of error of ± 5.6%, a confidence level of 95%, and a variance of 50%, we obtained 384 valid questionnaires as the sample size. The data were examined in two stages to achieve the objectives set. First, factor analysis was carried out to reduce the items to a smaller number of factors that facilitated the interpretation of the results. Specifically, a Varimax rotation method was used to minimize the number of variables that had high loads on each factor. In addition, the KMO index (Kaiser-Meyer-Olkin) and Bartlett’s test of sphericity were used to determine if the factor analysis model was appropriate. In the second stage, the multiple regression technique was used to select the dimensions of perceived value that predicted the variables of satisfaction and future behavior. Once the data were collected, they were organized, tabulated, and analyzed with the SPSS Version 22 program.

## 4. Results

### 4.1. Sociodemographic aspects of the sample

46.6% of the sample members were men, and 53.4% were women. Regarding marital status, 60.2% were single, and 33.3% were married. According to age, 50% were between 18 and 30 years old, and 22.9% were between 31 and 40. Furthermore, 62% had university studies, while 30.5% had secondary education. According to their occupation, 26.3% were private employees, 22.4% were students, and 17.4% were public employees. Moreover, 47.7% of the sample traveled with family, 23.7% traveled with friends, and 18.5% traveled alone. Finally, regarding their monthly income, 41.4% earned less than 500 USD, 38.8% earned between 500 and 1000 USD, and 15.1% made between 1000 and 1500 USD.

### 4.2. Perceived value in the cultural coastal destination

Factor analysis was performed to reduce the information of perceived value in a smaller number of factors. The varimax rotation method was used to sort the values into high and low. The factorial loads were greater than 0.5, which is appropriate for this type of study. Cronbach’s Alpha had values between 0.920 and 0.938, indicating a strong internal consistency in each factor. The KMO index was 0.90, showing a high value suitable for factor analysis. Bartlett’s test of sphericity was significant (p<0.01), so it was appropriate to perform the factor analysis (see Table 1).

**Table 1:** Perceived value in the cultural coastal destination (Factor analysis).

Variables	Emotional and social value	Economic value	Functional value
I make a good impression on other people	0.862		
I feel like a special person	0.811		
I get the social approval of others	0.726		
This visit makes me feel happy	0.691		
This visit is nice	0.689		
I have positive feelings	0.650		
The service is reasonably priced		0.898	
The service is economical		0.883	
The service offers good value for money		0.852	
The service is well organized			0.701
The service is convenient for me			0.651
The service has an acceptable level of quality			0.625
Cronbach’s Alpha	0.920	0.938	0.938
Variance explained (%)	59.242	14.301	6.296
Cumulative variance explained (%)	59.242	73.543	79.839

According to the results of Table 1, the first dimension was called *emotional and social value* because it was related to the emotion that tourists feel when visiting the coastal destination and the impression and social approval they want to have on other people. This dimension included 59.24% of the variance explained, making it the most salient factor compared to the others. The second dimension was related to economical services and good prices. The service is reasonably priced, indicating that the services were consistent with the income of tourists. The service in economical, which indicated that the services had low prices. And the service offers good value for money, which indicates that the services are worth the price paid for them. This dimension was assigned the name of *economic value*. It corresponded to 14.30% of the variance explained. On the other hand, the third dimension was called *functional value* since it was associated with organized and quality services. It included 6.3% of the variance explained. These results answer our first research question: RQ1: What are the dimensions of perceived value in cultural coastal destinations? Since three perceived values were found: emotional and social, economic, and functional.

### 4.3. Perceived value and demand satisfaction in the cultural coastal destination

The multiple regression method was used to analyze the predictors of demand satisfaction in coastal destinations (see Table 2).

**Table 2:** Perceived value and demand satisfaction.

Variable	Beta	t	Sig.	Tolerance
Emotional and social value	0.371	8.274	0.000	1.000
Economic value	0.214	4.776	0.000	1.000
Functional value	0.233	5.200	0.000	1.000
(Constant)		136.981	0.000	
F	39.435			
Sig.	0.000			
Durbin-Watson	1.897			

According to Table 2, the F test obtained significant values (p<0.05), indicating a relationship between the predictors (perceived value) and the response variable (demand satisfaction). The tolerance values did not reveal multicollinearity between the variables (tolerance as close as possible to 1). In addition, the Durbin-Watson statistic reached a value of 1.98 (within the range of 1.5 and 2.5), indicating no autocorrelation in the errors. These findings show that the emotional and social value, with a beta value of 0.371, was the most important predictor of tourists’ satisfaction. This means that tourists’ satisfaction is more influenced by emotional and social values. Therefore, if improvements are made in the emotional and social component of services, there will be an increase in the level of satisfaction of tourists in the coastal destination. These results answer our second research question: RQ2: What dimensions of perceived value predict demand satisfaction in cultural coastal destinations? This shows that the emotional and social value dimension primarily predicts the satisfaction of tourists who visit coastal destinations.

### 4.4. Perceived value and return to the coastal destination

Multiple regression was used as a predictive technique to analyze tourists’ intention to return to the coastal destination (see Table 3).

**Table 3:** Perceived value and return intentions.

Variable	Beta	t	Sig.	Tolerance
Emotional and social value	0.412	9.346	0.000	1.000
Economic value	0.207	4.693	0.000	1.000
Functional value	0.221	5.023	0.000	1.000
(Constant)		114.992	0.000	
F	44.872			
Sig.	0.000			
Durbin-Watson	1.738			

According to Table 3, the F test was significant (p<0.05), showing a relationship between the predictors and return intentions. The tolerance values (close to 1) revealed no multicollinearity between the variables. In addition, the Durbin-Watson statistic (value within the range of 1.5 and 2.5) showed no autocorrelation in the errors. These results indicate that the emotional and social value (beta = 0.412) predicts the return intentions in greater intensity. This means that the return of tourists depends largely on the emotional and social value they perceive in the coastal destination.

### 4.5. The perceived value and recommendation of the coastal destination

The most important predictors of recommendation of the coastal destination were obtained using the multiple regression technique (see Table 4).

**Table 4:** Perceived value and recommendation of the coastal destination.

Variable	Beta	t	Sig.	Tolerance
Emotional and social value	0.467	11.269	0.000	1.000
Economic value	0.226	5.458	0.000	1.000
Functional value	0.280	6.768	0.000	1.000
(Constant)		148.279	0.000	
F	67.525			
Sig.	0.000			
Durbin-Watson	1.988			

According to Table 4, the F test was significant (p<0.05), showing a relationship between the predictors and the recommendation variable. As the tolerance value was close to 1, there was no multicollinearity between the variables. In addition, there was no autocorrelation in the errors since the Durbin-Watson statistic was found within the range of 1.5 and 2.5. These results indicate that the recommendation intentions are predicted in greater proportion by the emotional and social value (beta = 0.467). Therefore, if the emotional and social value of services is improved, tourists will recommend the coastal destination more.

### 4.6. Perceived value and word of mouth about the coastal destination

Multiple regression was used to find the most important predictors of word of mouth about the destination (see Table 5).

**Table 5:** Perceived value and word of mouth about the coastal destination.

Variable	Beta	t	Sig.	Tolerance
Emotional and social value	0.447	10.683	0.000	1.000
Economic value	0.207	4.947	0.000	1.000
Functional value	0.300	7.167	0.000	1.000
(Constant)		149.131	0.000	
F	63.322			
Sig.	0.000			
Durbin-Watson	1.868			

According to Table 5, the F test was significant (p<0.05), evidencing a relationship between the predictors and the variable word of mouth about the destination. Since the tolerance values were close to 1, there was no multicollinearity between the variables. In addition, the Durbin-Watson statistic revealed no autocorrelation in the errors. These results show that the emotional and social value, with a beta value of 0.447, was the most salient predictor of word of mouth about the destination. Hence, if the emotional and social value of services is improved, tourists will spread more positive things about the coastal destination. These results answer our third research question: RQ3: What dimensions of perceived value predict loyalty variables such as return, recommendation, and word-of-mouth intentions about the cultural coastal destination? It is established that emotional and social value is the most salient predictor of loyalty in coastal destinations.

## 5. Discussion

The first objective of this study was to establish the dimensions of perceived value in a cultural coastal destination. The results show three dimensions: *emotional and social value*, *economic value*, *and functional value*. These findings are consistent with previous studies. The emotional and social value together was described by Carvache-Franco et al. [22] as emotional and social. Similarly, Kim and Thapa [21] found social value in a single dimension. Likewise, Schoeman et al. [20] identified a factor called perceived emotional value. The second dimension, economic value, was similarly found by Carvache-Franco et al. [23] as economic value. Also, Carvache-Franco et al. [22] established it together with another value, forming the economic-functional factor. The last dimension, functional value, was found analogously by Schoeman et al. [20] as perceived functional value. The contribution of the present study to the literature is to have found a dimension called emotional and social value that unifies two perceived values. It is also to have found the economic and the functional values separately. These finds have been found in a coastal destination with cultural attractions. This being the first study that identifies the dimensions of the perceived value in a destination with these characteristics.

The second objective was to determine the relationship between perceived value and satisfaction in a cultural coastal destination. The results show that the emotional and social value is the most important predictor of tourists’ satisfaction in coastal destinations. Different findings have been found in the literature. For example, Carvache-Franco et al. [23] found the emotional and functional dimensions as the most important for satisfaction. Also, Carvache-Franco [22] identified the economic-functional dimension as the one that most influences satisfaction. Other scholars have claimed that perceived value exerts a direct effect on satisfaction, such as Jin et al. [26], Hasan et al. [28], Hasan et al. [31]. The contribution of the present study is to have found that the emotional and social dimension is the main predictor of satisfaction in cultural coastal destinations, which had not been identified in previous studies.

The third objective of this research was to identify the relationship between perceived value and loyalty in variables such as return, recommendation, and word-of-mouth intentions in a cultural coastal destination. The results show that the emotional and social value is the most salient predictor of return, recommendation, and word-of-mouth intentions about the coastal destination. In previous findings, Carvache-Franco [22] focused on the emotional and social dimension as the most important predictor of loyalty in coastal and marine destinations. The contribution of the present study is to ratify the emotional and social value as the primary predictor of loyalty in cultural coastal destinations.

The results of this research will serve tourism service providers and the community so that they can develop products according to the characteristics of the demand. As practical implications, this study recommends coastal destination managers increase the emotional and social component by performing activities such as beach events, recreational activities and games, workshops to learn about water sports, contests, and festivals. For this reason, it is recommended to create programs for tourists to come in groups and to have group dynamics so that they can get to know each other by doing social activities together. Also, improving the services and customer service is essential to make tourist visits more pleasant. In addition, celebrating events such as birthdays in food and beverage establishments would increase the social dimension. The community must also be able to make events for tourists, such as craft-making workshops, teaching typical gastronomy, and experiential tourism activities. Sightings of marine flora and fauna would be another good option to increase coastal tourists’ social and emotional dimension Finally, teaching about the protection of the coastal environment is also recommended to increase the tourists’ emotional component in these coastal destinations.

## 6. Conclusions

Coastal tourism offers a range of activities ideal for the recreation of tourists, such as water sports, sightings of flora and fauna, visits to communities, and recreation on the beach. Perceived value involves evaluations of the benefits obtained in the travel experience, compared to the sacrifices made and conditioned by rational, affective, and social nature aspects. Perceived value is a multidimensional construct that starts through several factors that in the academic literature are not yet clear. Therefore, this study was conducted to determine the dimensions that constitute the perceived value in coastal destinations with cultural potential. The results show three dimensions of perceived value in cultural coastal destinations such as the case of Huanchaco in Peru: “emotional and social”, “economic”, and “functional” values. Of these, the emotional and social value is the most important predictor of tourists’ satisfaction, return, recommendation, and word-of-mouth intentions about the destination. Hence, if the emotional and social part of the services is improved, the level of satisfaction and loyalty of tourists in coastal destinations will increase. Finding together the emotional and social value could be due to the influence of cultural attractions to give greater importance to the social and emotional part of the tourist, so this social and emotional dimension becomes the main predictor of satisfaction and loyalty in the destinations. coasts with potential in cultural attractions. This will benefit the destination and the community, with greater return and recommendation intentions by tourists.

As theoretical implications, in cultural coastal destinations, it was shown that the perceived value is multidimensional and has three dimensions in coastal destinations. This study contributes to the academic literature by finding the emotional and social value as one dimension, which had only been found in one previous study, and identifying the economic and functional dimensions separately. Likewise, when analyzing the most salient dimension of satisfaction and loyalty, the results report that the emotional and social value exerts a positive influence. It is also reported in the academic literature that due to the importance of cultural attractions in this type of coastal destinations, it is possible to find the emotional and social as the main dimension of perceived value, whose influence predicts satisfaction and loyalty in cultural coastal destinations.

As practical implications, these results will help tourism service providers and the community because they will know the demand more clearly. Also, the administrators of tourist destinations can apply the present findings as management guides, and scholars can use them as a reference in the coastal tourism literature.

There are three major limitations in this study that could be addressed in future research. First, since the data were gathered during August and September, the results may be limited due to seasonal effects, considering that the demand can vary in other seasons. Another limitation was the non-probability convenience sampling method used since only tourists closest to the interviewers were surveyed. A third limitation was that the study only measured general satisfaction and satisfaction for each service at the destination was not measured. Finally, it is recommended as a future line of research to study the sociodemographic aspects that influence perceived value in coastal destinations.

## Supporting information

S1 File (SAV)

S2 File (SAV)
